# Older patients with heart failure managed in primary care versus cardiology care: a register-based study

**DOI:** 10.3399/BJGP.2025.0044

**Published:** 2025-10-21

**Authors:** Eric Chen, Mozhu Ding, Karolina Szummer, Monica Bergqvist, Karin Modig, Katharina Schmidt-Mende

**Affiliations:** 1 Academic Primary Health Care Centre, Stockholm Region, Stockholm, Sweden; 2 Division of Family Medicine and Primary Care, Department of Neurobiology, Care Sciences and Society, Karolinska Institute, Stockholm, Sweden; 3 Unit of Epidemiology, Institute of Environmental Medicine, Karolinska Institute, Stockholm, Sweden; 4 Department of Medicine, Karolinska Institute, Stockholm, Sweden

**Keywords:** cardiology care, drug therapy, heart failure, primary health care, Sweden, sociodemographic status

## Abstract

**Background:**

Adherence to guideline-recommended drug treatment for heart failure (HF) is lower among patients managed in primary care compared with cardiology care. Understanding more about the patient group managed in primary care only is important.

**Aim:**

To compare the sociodemographic characteristics, comorbidities, care use, and drug dispensation of older patients with HF managed exclusively in primary care with those who are also managed in cardiology care.

**Design and setting:**

A register-based study using real-world administrative data from Stockholm, Sweden.

**Method:**

The study population comprised all individuals aged ≥60 years resident in Stockholm on 31 December 2022 with an HF diagnosis. The Total Population Register and several national health registers were linked, providing information on comorbidities, HF hospital admissions, primary care visits, and dispensed drugs. Individuals were categorised into those managed exclusively in primary care or those who were also managed in cardiology care by the absence/presence of an in-/outpatient appointment with a cardiologist during the past 5 years.

**Results:**

HF was prevalent in 33 872 of 524 250 (6.5%) individuals of whom 50.4% (*n* = 17 067) were exclusively managed in primary care. Among patients also managed in cardiology care, two-thirds of HF drugs were prescribed by primary care. Primary care-managed patients were on average 3 years older, more often female, of lower socioeconomic status, had fewer comorbidities, received fewer guideline-recommended drugs, and had lower rates of admissions to hospital for HF than cardiology care-managed patients. Residing in a nursing home and having dementia were the factors most strongly associated with exclusive primary care management.

**Conclusion:**

Primary care manages the majority of individuals with HF, who are typically older than those patients who are also managed in cardiology care. These characteristics may explain differences in drug use.

## How this fits in

Patients with heart failure (HF) managed by primary care receive guideline-recommended drug treatment less frequently than patients seeing cardiologists, for reasons only partly understood. The findings of the current study highlight the major role primary care plays in managing patients with HF, with patients managed only in primary care typically older, female, and of lower socioeconomic status. Awareness of these differences will allow clinicians to make conscious decisions on drug treatment that are beneficial to this population.

## Introduction

Heart failure (HF) is a growing global health concern, with current prevalence estimates ranging from 1% to 3% in the general adult population^1^ and as high as 5%–13% among individuals aged ≥60 years.^2^ HF leads to increased healthcare use, higher mortality, reduced quality of life, and a significant economic burden worldwide.^3^

A cornerstone of HF treatment is the use of drugs^4^ that have proven to reduce morbidity and mortality in patients with reduced ejection fraction (EF, ≤40%).^5^ However, several studies have shown a gap between ideal and real-world prescription patterns, for example, a gap of 13% for angiotensin-converting enzyme inhibitors (ACEi)/angiotensin receptor blockers (ARBs) and 17% for mineralocorticoid receptor antagonists (MRAs) in overall HF populations.^6^ Older age, female sex, and presence of comorbidities have been associated with larger treatment gaps.^7,8^ Furthermore, gaps are larger for patients managed in primary care than in cardiology care.^9–11^

Past research suggests that patients with HF managed in primary care are highly burdened by comorbidities and polypharmacy, which may complicate and potentially impede prescribing according to guidelines.^12^ However, studies comparing patients with HF managed in primary care and cardiology care are sparse. Two studies using the Swedish HF Register (SwedeHF) show that patients planned for follow-up in primary care are older, more frequently female, have a greater burden of comorbidities, have lower educational levels and income, and are less likely to receive HF medications according to guidelines.^9,13^ However, these studies analysed only a subset of patients with HF managed in primary care, leaving it uncertain how patients managed in primary care differ from those managed in cardiology care.

Given the expected rise in prevalence of HF, the increased importance of primary care as a hub in health care, and seemingly insufficient quality of care for HF in primary care, it is crucial to better understand characteristics of patients with HF who are managed in primary care. Sweden is known for comprehensive and high-quality health registers, which presents an excellent possibility of addressing this knowledge gap. Using primary care and cardiology care data from the entire population of Stockholm, Sweden, the study aimed to describe an HF population in terms of sociodemographic status, comorbidities, healthcare use, and dispensed HF medications, stratified by management in primary and cardiology care.

## Method

### Study population

The study population comprised all individuals aged ≥60 years residing in Stockholm, Sweden, on 31 December 2022, with an HF diagnosis anytime between 2013 and 2022. They were identified in the Total Population Register. To ensure reliable estimates of comorbidities and healthcare use, individuals were required to have a continuous 5-year residency before 31 December 2022 in Stockholm. Through a unique personal identification number assigned to all individuals registered in Sweden, the Total Population Register is linked to individual-level data from the Stockholm Region’s Central Data Warehouse (VAL), a database containing all primary care visits and diagnoses of 2.4 million inhabitants of Stockholm,^14^ and the National Patient Register (NPR) containing data on inpatient and outpatient secondary health care.^15^ In this study, HF diagnoses were identified as a visit in primary or specialised cardiology in- or outpatient care with a diagnosis code of I50, excluding I50.0 (right-ventricle HF) according to the International Classification of Diseases, 10th revision (ICD-10). Starting from January 2021, ICD-10 codes specifying EF were available: reduced EF ≤40% (HFrEF), midrange EF 41%–49% (HFmrEF), and preserved EF ≥50% (HFpEF). When available, these codes were prioritised over HF codes that did not specify EF, allowing more precise characterisation of HF subtypes within the population.

### Sociodemographic variables

Sociodemographic data were obtained from the Longitudinal Integrated Database for Health Insurance and Labor Market Studies, with information on date/country of birth, sex, highest level of education, and family disposable income. Country of birth was dichotomised as born in versus outside of Sweden. Educational level was categorised into elementary school (≤9 years), high school (10–12 years), and university or higher (≥13 years*).* Information on cohabitation status was retrieved from the Swedish Dwelling Register.

### Comorbidities

Twelve comorbidities relevant to quality of drug treatment in patients with HF were identified using ICD-10 codes (see Supplementary Table S1). These codes were traced across all healthcare contacts in the NPR and VAL from 2018 to 2022 and treated as a dichotomous variable. Additionally, the Hospital Frailty Risk Score^16^ and the Charlson Comorbidity Index^17^ were calculated using NPR and VAL data.

### Care utilisation

In Stockholm, patients with HF are managed in primary care by GPs and district nurses. Primary care has access to laboratory testing, electrocardiograms, and echocardiography for the assessment of HF. New cases of patients with HF, patients with pharmacologically optimised but symptomatic HF, or patients with complicated cases who had cardiac or non-cardiac comorbidities are referred to cardiologists, who refer patients back to primary care when stabilised.

Individuals with HF managed exclusively by primary care were defined as those with no recorded contact with a cardiologist (an HF diagnosis related to an outpatient visit or inpatient stay in a cardiology clinic) in the past 5 years. Conversely, individuals also managed in cardiology care were defined as those with at least one contact with a cardiologist in the same time period. The total number of visits to a primary care physician per individual, regardless of diagnosis, between 2018 and 2022 was reported.

Data on admissions to hospital because of HF were obtained from the NPR. Hospital admissions with HF as either the primary or secondary diagnosis were recorded per individual. Hospital admissions lasting ≥24 h in cardiology, internal medicine, or geriatrics departments were included based on a sensitivity analysis showing that 93.8% of all admissions to hospital with HF diagnosis occur at these clinic types.

Information on long-term care (home care or nursing home) came from the Social Service Register.^18^ ‘Home care’ refers to all publicly funded social services support provided at home, excluding medical care, whereas 'nursing home' refers to institutional care settings. Furthermore, the study identified individuals with home health care, which refers to healthcare services provided by primary care. This information was retrieved from the VAL and defined as at least one registered contact from 2018 to 2022.

### Drug dispensing

Data on drug dispensing for six standard HF medications were extracted from the Swedish Prescribed Drug Register^19^ using Anatomical Therapeutic Chemical codes. The medications analysed were angiotensin receptor neprilysin inhibitors (ARNIs), C09*D*×04, ACEi/ARBs, C09 except C09*D*×04, beta-blockers (BBs), C07, MRAs, C03DA, diuretics, C03CA, and sodium-glucose cotransporter 2 inhibitors (SGLT2is), A10BK. The analysis covered the year of 2022. Additionally, the clinic type (primary care or specialised care) associated with the last recorded dispensation of each medication was noted.

### Statistics

Data were presented as mean values and standard deviations (SDs) for normally distributed variables, and medians with interquartile ranges (IQRs) for non-normally distributed variables. Categorical variables were compared using the chi-squared (χ²) test. For continuous variables, the Student’s *t*-test was used for normally distributed data, while the Mann–Whitney *U*-test was applied for non-normally distributed data. A two-sided *P*-value of <0.05 was considered statistically significant.

A logistic regression was performed to examine the association of demographic characteristics, comorbidities, and dispensed drugs with HF management in cardiology care. Results are reported as odds ratios (ORs) with 95% confidence intervals (CIs).

All analyses were conducted using RStudio (version 2023.12.1).

## Results

HF was identified in 33 872 out of 524 250 individuals aged ≥60 years in Stockholm as of 31 December 2022, representing a prevalence of 6.5%. Table 1 outlines the characteristics of the HF population, stratified by management exclusively in primary care or also in cardiology care. The mean age of the HF population was 79.6 years (SD 8.9), and 45.7% (*n* = 15 485) were female. Half of the population (*n* = 17 067, 50.4%) were managed exclusively in primary care. In general, individuals managed in primary care were older than those also managed in cardiology care (mean age 81.2 [SD 8.9] versus 78.0 [SD 8.6] years), more often female (51.5% versus 39.9%), had a lower educational level (university or above 43.0% versus 48.6%), were less likely to have a disposable income above median (45.5% versus 54.5%), and more likely to live alone (cohabitation 46.3% versus 53.6%), receive home care (22.9% versus 18.7%), or live in a nursing home (12.6% versus 5.7%). Most individuals with HF (79.1%) had an ICD-10 code lacking specification on EF. Individuals who had seen a cardiologist were more likely to have a diagnosis specifying EF, particularly HFrEF.

**Table 1.** Characteristics of the HF population as of 31 December 2022 in Stockholm Region, stratified by managed exclusively in primary care versus those also managed in cardiology care

Characteristic	Total (*N* = 33 872)*n* (%)^a^	Primary care (*N* = 17 067, 50.4%)*n* (%)^a,b^	Cardiology care (*N* = 16 805, 49.6%)*n* (%)^a,c^
**Age, mean (SD)**	79.6 (8.9)	81.2 (8.9)	78.0 (8.6)^d^
**Age group, years**			
60–69	5027 (14.8)	1914 (11.2)	3113 (18.5)^d^
70–79	11 548 (34.1)	5232 (30.7)	6316 (37.6)^d^
80–89	12 492 (36.9)	6697 (39.2)	5795 (34.5)^d^
≥90	4805 (14.2)	3224 (18.9)	1581 (9.4)^d^
**Sex, female**	15 485 (45.7)	8784 (51.5)	6701 (39.9)^d^
**Born outside of Sweden**	7977 (23.6)	4127 (24.2)	3850 (22.9)^d^
**Education^e^**	33 034	16 560	16 474
Elementary school	8982 (27.2)	4881 (29.5)	4101 (24.9)^d^
High school	8915 (27.0)	4556 (27.5)	4359 (26.5)
University or above	15 137 (45.8)	7123 (43.0)	8014 (48.6)^d^
**Disposable income above median**	16 936 (50.0)	7774 (45.5)	9162 (54.5)^d^
**Cohabitation**	16 899 (49.9)	7897 (46.3)	9002 (53.6)^d^
**Home care^f^**	7051 (20.8)	3913 (22.9)	3138 (18.7)^d^
**Nursing home^f^**	3113 (9.2)	2149 (12.6)	964 (5.7)^d^
**Home health care^f^**	8399 (24.8)	4655 (27.3)	3744 (22.3)^d^
**Visits to primary care per year, median (IQR)**	4.6 (2.4–8.4)	4.8 (2.4–8.6)	4.6 (2.4–8.4)
**HF subtype**			
HFrEF	3507 (10.4)	320 (1.9)	3187 (19.0)^d^
HFmrEF	1343 (4.0)	234 (1.4)	1109 (6.6)^d^
HFpEF	2236 (6.6)	573 (3.4)	1663 (9.9)^d^
Unspecified HF	26 786 (79.1)	15 940 (93.4)	10 846 (64.5)^d^
**Hospital Frailty Risk Score, median (IQR)**	4.4 (1.1–10.8)	4.4 (0.9–11.1)	4.4 (1.1–10.4)
**Hospital Frailty Risk Score groupings**			
Low (<5)	16 109 (47.6)	8116 (47.6)	7993 (47.6)
Moderate (5–15)	12 426 (36.7)	6106 (35.8)	6320 (37.6)
High (>15)	5337 (15.8)	2845 (16.7)	2492 (14.8)
**Charlson Comorbidity Index, median (IQR)**	2 (1–4)	2 (1–4)	3 (2–5)^d^

^a^Data are *n* (%) unless otherwise specified. ^b^Primary care management is defined as the absence of a physical visit to a cardiologist in the past 5 years, 1 January 2018 to 31 December 2022. ^c^Cardiology care management refers to at least one cardiology contact in the past 5 years. ^d^ *P*<0.05 between groups. ^e^Missing data in education account for 2.5%. ^f^Prevalence of home care in general population adults >60 years is 5.8% and in nursing homes 2.9%. EF = ejection fraction. HF = heart failure. HFmrEF = HF with midrange EF 41%–49%. HFpEF = HF with preserved EF ≥50%. HFrEF = HF with reduced EF ≤40%. IQR = interquartile range. SD = standard deviation.

**Figure 1.**
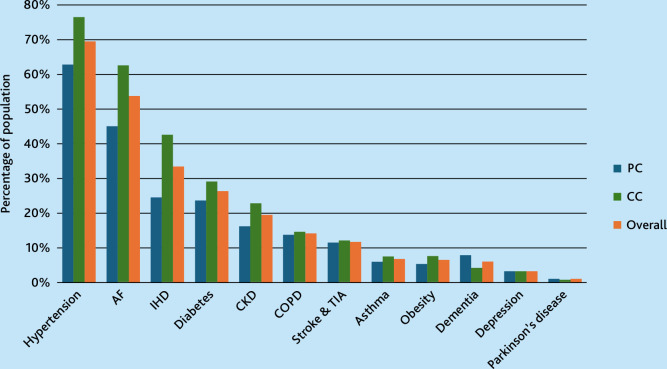
Prevalence of coexisting diseases in patients with heart failure, stratified by managed exclusively in primary care (PC) versus those also managed in cardiology care (CC). Coexisting diseases defined as any diagnosis during the past 5 years. Statistical significant difference exists for all variables except for ‘Stroke & TIA’ and ‘Depression’; *P*<0.05. AF = atrial fibrillation. CKD = chronic kidney disease. COPD = chronic obstructive pulmonary disease. IHD = ischaemic heart disease. TIA = transient ischaemic attack.

The number of comorbidities differed between individuals managed in primary care versus cardiology care. Individuals managed in primary care had a lower prevalence for most comorbidities (Figure 1). However, dementia and Parkinson’s disease were more prevalent among individuals treated in primary care only compared with those also managed in cardiology care (dementia 8.0% [*n* = 1366] versus 4.3% [*n* = 726], and Parkinson’s disease 1.2% [*n* = 202] versus 0.8% [*n* = 142]).

**Figure 2.**
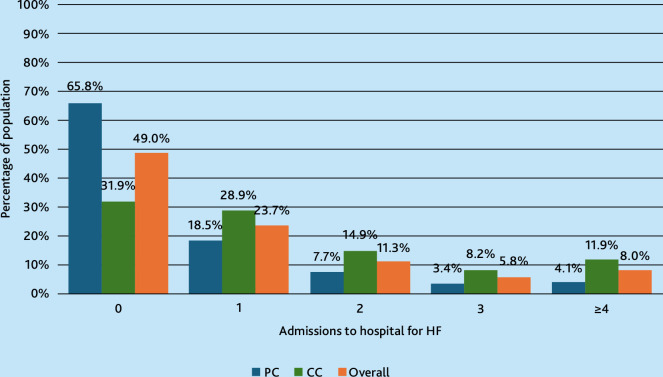
Number of admissions to hospital for heart failure (HF) in the past 5 years (2018–2022), stratified by managed exclusively in primary care (PC) versus those also managed in cardiology care (CC).

Admissions to hospital for HF are presented in Figure 2. Half of the population (*n* = 17 277, 51.0%) had been admitted to hospital at least once for HF in the past 5 years. Individuals managed in primary care were less likely to be admitted to hospital for HF compared with those also managed in cardiology care (34.2% [*n* = 5841] versus 68.1% [*n* = 11 436]). No significant difference was observed between the two groups in terms of frequency of visits to primary care physicians.

**Figure 3.**
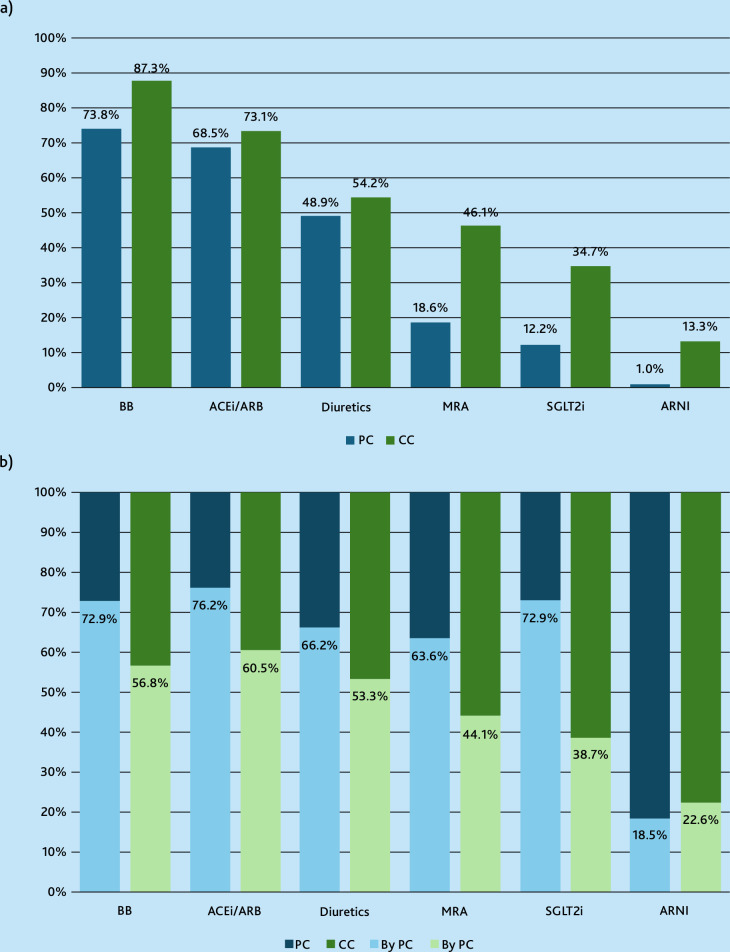
a) Proportion of patients with heart failure (HF) with specific types of HF medications stratified by managed exclusively in primary care (PC) versus also in cardiology care (CC). Statistically significant difference between the groups existed across all drug classes; *P*<0.05. b) Proportion of HF medication prescriptions that were made by primary care (lighter colour) versus outside of primary care, stratified by management in primary care (blue) and cardiology care (green). ACEi = angiotensin-converting enzyme inhibitor. ARB = angiotensin II receptor blocker. ARNI = angiotensin receptor neprilysin inhibitor. BB = beta-blocker agent. MRA = mineralocorticoid receptor antagonist. SGLT2i = sodium-glucose cotransporter 2 inhibitor.

Figure 3a shows that individuals managed in primary care were dispensed medications from all HF drug classes less frequently compared with those also managed in cardiology care. In general, BBs and ACEi/ARBs were the most dispensed drugs, 80.5% (*n* = 27 274) and 70.8% (*n* = 23 975), respectively; whereas ARNIs were dispensed to only 7.1% of the population, and almost exclusively to those managed by cardiology care. Figure 3b illustrates the origin of the last HF drug prescription and primary care accounted, on average, for 60.0% (*n* = 53 979/89 959) of all dispensed HF drugs, regardless of whether individuals were managed in primary care only or also in cardiology care.

**Figure 4.**
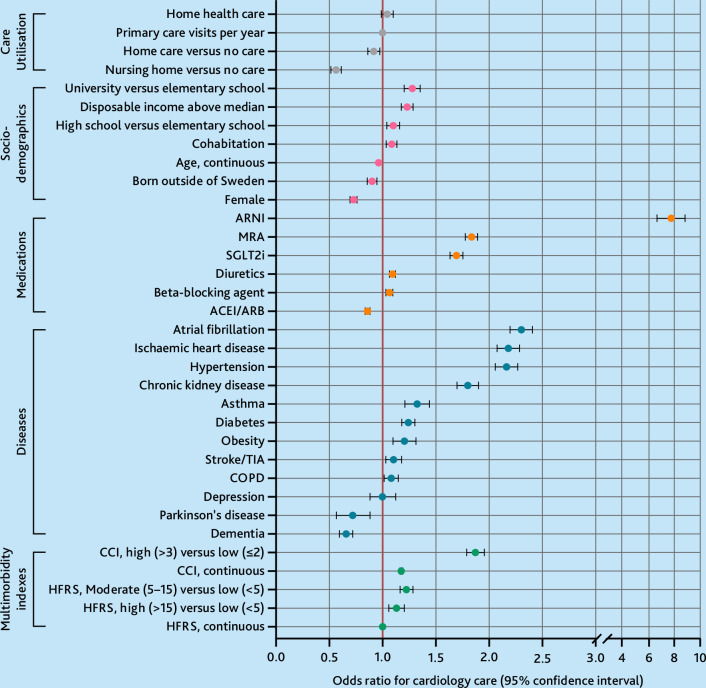
Odds ratios with 95% confidence intervals for seeing a cardiologist in the past 5 years (2018–2022) by number of predictive variables. Analyses adjusted for age and sex. Categorised by variable type. ACEi = angiotensin-converting enzyme inhibitor. ARB = angiotensin II receptor blocker. ARNI = angiotensin receptor neprilysin inhibitor. BB = beta-blocker agent. CCI = Charlson Comorbidity Index. COPD = chronic obstructive pulmonary disease. HFRS = Hospital Frailty Risk Score. MRA = mineralocorticoid receptor antagonist. SGLT2i = sodium-glucose cotransporter 2 inhibitor. TIA = transient ischaemic attack.

Figure 4 shows age- and sex-adjusted ORs for seeing a cardiologist the past 5 years by number of predictive variables. ARNI, atrial fibrillation, ischaemic heart disease, and hypertension were most associated with increased odds of seeing a cardiologist. Long-term care was associated with a reduced odds of seeing a cardiologist; nursing home residency (OR 0.56, 95% CI = 0.52 to 0.61) and home care (OR 0.91, 95% CI = 0.87 to 0.97). Among comorbidities, dementia (OR 0.66, 95% CI = 0.60 to 0.72) and Parkinson’s disease (OR 0.71, 95% CI = 0.57 to 0.89) were associated with reduced odds of seeing a cardiologist.

## Discussion

### Summary

This large population-based study found that half of individuals aged ≥60 years with HF are managed exclusively by primary care, with no cardiologist contact over 5 years. Moreover, primary care is responsible for 60.0% (*n* = 53 979/89 959 prescriptions) of the overall prescribing of HF medications, regardless of management in primary care or cardiology care. Individuals managed exclusively in primary care are on average 3 years older, more often female, of lower socioeconomic status, and more likely to receive home care or reside in a nursing home. Dementia and Parkinson’s disease are more prevalent among individuals seen only in primary care. Larger drug treatment gaps for all drug classes were observed for primary care versus cardiology care patients.

### Strengths and limitations

A strength of the study is the linkage across multiple databases including primary and secondary care registers, which enables a comprehensive and detailed description of patient characteristics, healthcare use, and drug dispensation in a real-world HF population. Dispensed as opposed to prescribed drugs were analysed, allowing more accurate representation of actual medication use.^19^ Furthermore, the high-quality Swedish registers enable coverage of the entire HF population in Stockholm without selection.

However, there are several limitations. The study defined management in primary care and cardiology care using an arbitrary, but rather long, 5-year cut-off based on presence or absence of a cardiologist contact. This approach may not fully capture the complexity of care pathways. For instance, management in cardiology care would not have captured written recommendations given by cardiologists or patients followed exclusively by HF nurses at cardiology clinics without meeting a cardiologist.^20^ However, the authors believe that these circumstances are exceptional, and this possible source of bias is negligible. Moreover, the results revealed that the primary care group seems to be heterogeneous, consisting of both the oldest-old and the younger and healthier without the need of cardiology care. This likely suppressed absolute differences between groups.

Clinical data, such as echocardiography and New York Heart Association class, and laboratory data, such as creatinine, were unavailable, limiting the ability to describe severity of HF and comorbidities in more detail. This is of particular interest because the physician’s compliance to prescribe recommended HF drugs strongly relies on EF and comorbidities such as renal dysfunction. Such data would also increase the validity of HF diagnoses. A study using the National Patient Register showed that the overall validity of HF diagnosis was 82%, which was further improved to 88% when supported by an echocardiography.^21^ This small difference would be unlikely to change the current findings significantly. Furthermore, the results may underestimate the prevalence of HF and comorbidities because of incomplete coding by healthcare providers.

### Comparison with existing literature

Research on who provides care to older patients with HF is sparse. A study using the SwedeHF found that 36% of patients with HF were followed up in primary care,^9^ whereas a study by Zarrinkoub *et al* found only 30% of patients with HF were managed in primary care.^22^ The current findings show a higher proportion of management only in primary care (50%), although these numbers are difficult to compare as they depend on how the subpopulations are defined. The current study focused on describing patients with HF managed exclusively in primary care, with no appointment with a cardiologist in an in-/outpatient setting over 5 years. Therefore, a portion of patients with HF in the cardiology care group might be incorrectly classified, for example, because of an HF-related admission to hospital 4 years ago. If anything, the current study underestimates the proportion of patients with HF managed in primary care.

In contrast with former studies, individuals in the cardiology care group had a higher comorbidity index compared with those in the primary care group,^9,23^ which may be explained by different data sources. The SwedeHF register includes nationwide data, covering more rural areas in Sweden where care by cardiologists is less available, thus reliance on primary care greater. Therefore, primary care manages patients with a greater number of comorbidities.^24^ Additionally, regional factors such as greater use of specialty care in Stockholm^25^ and disparities in socioeconomic status^26^ may contribute to these differences.

The current findings confirm that primary care management is associated with older age and female sex.^9,27^ The association of primary care management with dementia and living in a nursing home has recently been described in a Japanese population;^28^ however, a novelty in the current study is the association with Parkinson’s disease. This aligns with the findings from a review concluding that patients with dementia may not have the same access to treatment for comorbidities as those without dementia.^29^

The current study found larger HF drug treatment gaps between patients managed in primary care versus those also managed in cardiology care than previously reported (ACEi/ARB/ARNI 16.9% [*n* = 2647] versus 11.2%, BBs 13.5% [*n* = 2084] versus 6.8%, MRA 27.5% [*n* = 4581] versus 11.5%).^9^ The smaller gap in Lindberg and colleagues’ study could be a selection bias, as participation in SwedeHF by primary care practices is voluntary, with those demonstrating effective HF management possibly more inclined to participate. The current study adds to existing literature by describing the source of last prescription dispensation. In the current study, most prescriptions were issued in primary care, which could be explained by cardiologists providing the initial treatment recommendation, whereas primary care repeats prescriptions of HF medications. To the authors' knowledge, this detail has never been reported before.

### Implications for research and practice

Primary care plays a major role in the management of older adults with HF, particularly the oldest individuals receiving long-term care. This likely explains why drug treatment gaps in patients with HF managed only in primary care are larger. However, patients with HF with characteristics like those managed in primary care are unlikely to be included in the randomised controlled trials underpinning current guidelines.^30^ It is questionable whether guidelines are applicable to patients with HF who are managed in primary care. Meanwhile, there is not a single GP among the 31 authors of current European HF guidelines.^4^ The findings in the current study call for a greater role for GPs in the development of HF guidelines. Future research should complement traditional outcome measures in HF, such as cardiovascular mortality, admissions to hospital for HF, and achieved target doses, with more patient-centred outcomes, including quality of life and individual treatment goals, particularly in populations with HF who are older and with multimorbidity.^31^

## Data

The datasets analysed during the current study are not publicly available because of the General Data Protection Regulation in Sweden. Access to the data and the codes for data analyses can be permitted to external researchers after ethical vetting and establishment of a collaboration agreement. The corresponding author should be contacted for questions about data sharing.
